# The Association between Parental Support and Adolescents’ Psychological Complaints: The Mediating Role of a Good School Climate

**DOI:** 10.3390/children8070550

**Published:** 2021-06-25

**Authors:** Joacim Ramberg

**Affiliations:** Department of Special Education, Stockholm University, S-11419 Stockholm, Sweden; joacim.ramberg@specped.su.se

**Keywords:** psychological complaints, mental health problems, parental support, school climate, mediating, adolescence

## Abstract

Parental support is an important factor affecting young people’s mental well-being, but the school climate also plays an important role. However, few studies have previously examined whether the school climate serves as a mediator for adolescents’ mental health problems. This study aimed to investigate the association between parental support and students’ psychological complaints, while also examining the possible mediating role that a good school climate may have. Data derives from 5783 senior-level students (age 15–16) distributed over 152 school units in Stockholm municipality. Regression linear analysis was used for the analysis and Baron and Kenny’s four-step mediation model has been applied. Sobel’s test was conducted in order to test the significance of the mediation effect. The results show that there is a significant negative association between parental support and students’ psychological complaints, and that school climate has a mediating role in this association. It can be concluded that school climate has a partly mediating role in the association between parental support and students’ psychological complaints. Therefore, it seems important to develop the school climate in order to strengthen this source of support to reduce mental health problems among adolescents.

## 1. Introduction

Mental health problems and psychological complaints are common during adolescence, and the problems have increased in many countries, including Sweden, with girls in particular reporting high levels of psychological complaints [1,2]. Research indicates that up to one in five children globally experience mental health problems [3], which is why it is one of the World Health Organization’s (WHO) top priorities when it comes to promoting global health [4]. In addition to the negative consequences mental health problems have for young people, it may also persist into adulthood and thus have negative consequences throughout the life course [2,5].

Pathways to good health, well-being, and positive social function later in life have their roots in childhood and adolescence [6,7,8]. At this stage of life, the relationship to parents is crucial for young people’s well-being [9]. The quality of parenthood is thus an important factor affecting young people’s emotional well-being, and in the same way, it also affects the degree of psychological complaints.

Based on this, increasing knowledge and efforts concerning what can make parents more supportive and involved in their children’s life emerges as important. Regardless of this, there will always be children who will grow up without parental support or with low levels of parental support, so it also appears important to look for other factors or protective environments that can counteract the effects of low or non-existent parental support. One such environment may be the school, in which the vast majority of children and adolescents spend a significant part of their time. Previous research has shown that support from both parents and adults working in the school is important for young people’s health and well-being [10,11,12], and that these two sources of support to some extent are independently associated with young people’s health and well-being [13,14]. School is a particularly important context for young people as most spend almost half of their waking time there. This means that a large part of young people’s social relationships take place within the school, and therefore, the school climate is a particularly important factor to investigate in relation to young people’s well-being. However, there has been less investigation of whether a good school climate can compensate for low parental support in terms of psychological complaints. It is conceivable, for example, that young people who have low or non-existent support from their parents to a greater extent seek support in other contexts, such as school, and that the school could then to some extent compensate for low parental support.

The school’s climate has for a long time been recognized as an important factor that contributes to shaping children and young people [15]. However, as stated in previous research, little is known about the mediating role of school climate on the effects of psychological health [16]. Thus, this study seeks to investigate the possible mediating role that a good school climate can have in the association between parental support and psychological complaints among adolescents in the context of Stockholm municipality, Sweden. To this end, data collected in 2014 and in 2016 from 5783 students in 152 school units in Stockholm municipality have been used.

### 1.1. Psychological Complaints

Psychological complaints include a variety of states of mind related to individual well-being, such as feelings of sadness, depressed mood, fear, sluggishness, anxiety, and general feelings of reduced well-being [17,18]. Just like in many other countries, there has been an increase in health complaints among adolescents in Sweden in recent decades, and this increase seems to be general to all different groups in society [5,19]. The Public Health Agency of Sweden reports that the proportion of Swedish 13- and 15-year-old girls and boys reporting multiple health complaints has doubled since around 1985, and that the increase has been the same among boys and girls, even if a larger proportion of girls report multiple health complaints. It is also pointed out that the increase has been more pronounced in Sweden than in other Nordic countries [19], so examining possible protective factors in this national context is important. Globally, it has been reported that up to 20% of adolescents have mental health problems that affect their life situation negatively [3,20]. It is well-established in previous research that girls report psychological complaints more frequently than boys, and that this gender gap increases with age during adolescence [21,22,23].

### 1.2. Parental Support

Adolescents usually have extensive social networks and relationships that include, for example, peers, schoolmates, school staff, siblings, and parents, who can all serve as sources of support [24,25]. Previous research shows that parents are an important source of support during adolescence. Studies show, for example, that students experiencing more support from their parents also report fewer internalizing and externalizing problems [9,26,27,28,29,30]. Likewise, high levels of parental support have been shown to be associated with better self-esteem, social well-being, and lower depression among adolescents, just as low parental support has been shown to be linked to psychological distress and psychological complaints to a greater extent than found among those having high levels of parental support [30,31,32]. Previous research has also shown that a lack of parental support cannot fully be compensated by support from peers, meaning that those two sources of support are to some extent independent of each other [33]. Overall, it can be concluded that parental support plays a major role in young people’s well-being, and although relationships with peers are becoming increasingly important in young people’s lives, studies show that the important role of the family remains during adolescence [33,34].

### 1.3. School Climate

The concept of school climate is used in slightly different ways in the literature, but the term is often used to describe the school’s quality and character, which reflects rules, goals, values, interpersonal relationships, teaching and learning methods, and organizational structures within a school. A good school climate also means the school provides a sense of security, and that the relationships among the various participants are functional [35,36,37]. It is further described as involving elements such as social, emotional, and physical safety, the presence of respectful behavior among participants, a focus on learning, and collaboration among students, teachers, and families [12,15].

Previous studies have shown that a good school climate is associated with several positive outcomes among students, such as the promotion of psychosocial development [38], and reducing risk behaviors such as smoking, decreased alcohol use [39], and aggressive behavior [40]. A good school climate has also been linked to improved mental health outcomes and academic performance [41].

Taken together, as stated in a literature review by Aldridge and McChesney [15] concerning the relationship between school climate and adolescent mental health and well-being: “The findings of our review provide strong evidence of the importance of the school climate in influencing adolescents’ mental health” (p.135).

Overall, it can be stated that both parental support and a good school climate are important factors influencing young people’s well-being for the better, but there has been less investigation of whether a good school climate serves as a mediator in this relationship.

### 1.4. Aim

This study aims to examine the association between parental support and students’ psychological complaints, as well as to investigate the possible mediating role that a good school climate may have, while also taking a range of sociodemographic characteristics at the individual and the school level into account.

The study is based on the following hypotheses:

**Hypothesis** **1** **(H1).** *Students’ ratings of parental support are negatively associated with psychological complaints*.

**Hypothesis** **2** **(H2).** *This association remains when adjusting for sociodemographic characteristics at both the individual and the school level*.

**Hypothesis** **3** **(H3).** *A good school climate serves as a mediator in the relationship between parental support and psychological complaints*.

## 2. Materials and Methods

### 2.1. Data

The data used in this study derive from the Stockholm School Survey (SSS) of 2014 and 2016, targeting a sample of grade 9 students (aged 15–16 years) in all public and many independent schools in the Stockholm municipality. The SSS is carried out every two years by the Stockholm municipality and consists of a variety of questions on different subjects, and provides information on, for example, psychological complaints, parental support, school climate, and a range of sociodemographic characteristics. The survey was conducted during class with questionnaires administered by the teachers, with a response rate of 82.7 percent. The survey was also linked to school-level data from official records provided from the Swedish National Agency of Education (SNAE) [42]. Students with internal non-responses on any of the included variables were excluded, as were students attending a school with no school-level information from the SNAE, which resulted in a final study sample of 5783 students distributed over 152 school units. According to a decision by the Regional Ethical Review Board of Stockholm (2010/241-31/5), the data are not considered as an issue of ethical concern, since the data were collected anonymously.

### 2.2. Variables

#### 2.2.1. Dependent Variable

Psychological complaints is the dependent variable used, assessed by six items capturing different psychological complaints in terms of sadness, depressed mood, fear, insufficiency, inactivity, uneasiness, and absence of enjoyment of life. All items were rated by students on a five-point Likert-type scale and values from all items were added to form a sum index in the range 6–30, with higher scores indicating more psychological complaints. By using Confirmatory Factor Analysis (CFA), factor loadings for each item representing psychological complaints were produced, and the factor loadings range between 0.63 and 0.79. The index has a high internal consistency (Cronbach’s α = 0.80). All items and response options are presented in Table 1.

#### 2.2.2. Independent Variable

Parental support is the main independent variable and was created from five items in order to capture the overall parental support, as rated by students. The items covered issues such as praise from parents, encouragement, being noticed, care, and whether they perceive their parents as role models or not. All questions were responded to on a four-point Likert-type scale forming a sum index in the range 5–20, with higher scores indicating more parental support. The CFA showed factor loadings from 0.63 to 0.85, and the index had a high internal consistency (Cronbach’s α = 0.82). All questions and response options are presented in Table 1.

### 2.3. Possible Confounders (Individual Level)

Several variables at the individual level that may act as potential confounders were used in the analysis. Students’ grades were measured as the summation of the students’ self-reported grades in the subjects Swedish, English, and mathematics. Grades given in letters (A–F) were given numerical values (5–0), resulting in an approximately normally distributed index in the range 0–15. Gender was coded as boy or girl. Family structure was measured by asking ‘Which persons do you live with?’ where the students could mark one or several options. Those who ticked both ‘Mother’ and ‘Father’ were classified as living with two custodial parents in the same household, and contrasted to all others. Parental education was created from the question ‘What is the highest education your parents have?’ with the following response options to be ticked separately for the mother and the father: ‘Compulsory’, ‘Upper secondary school’, and ‘University’. The variable was coded into those having no parent with post-secondary education and those having at least one parent with post-secondary education. Time in Sweden was measured by the question ‘How long have you lived in Sweden?’ with the response options (a) ‘All my life’, (b) ‘10 years or more’, (c) ‘5–9 years’, and (d) ‘Less than 5 years’. This variable was dichotomized into those who had lived in Sweden for less than ten years and those who had lived in Sweden for ten years or more, thus roughly distinguishing those who had attended Swedish school throughout compulsory school from all the others. Year of data collection was also adjusted for in the analysis.

### 2.4. Possible Confounders (School Level)

At the school level, several potential confounders were included in the analysis. These data were retrieved from official records from the SNAE. School type refers to public or independent school. Proportion of full-time teachers per student indicates the student–teacher ratio at the school, proportion of full-time teachers with pedagogical education indicates the proportion of teachers at the school who have a pedagogical degree, while proportion of students with foreign background refers to the proportion of students born abroad and/or whose parents were both born abroad.

### 2.5. Potential Mediator

School climate is the potential mediator variable and consists of 18 items, rated by students, and captures dimensions such as positive attitudes towards school, involvement in decision-making, awareness of regulations and rules of procedure, praise and support from teachers, quiet working environment, and safety. All items were responded to on a four-point Likert-type scale forming a sum index in the range 18–72, with higher scores indicating a good school climate. The index had a high internal consistency (Cronbach’s α = 0.84). All questions and response options are presented in Table 1.

### 2.6. Statistical Method and Analytical Strategy

Linear regression analysis was used for the analysis and the formulated hypotheses were tested in a number of models. Model 1 targets the first hypothesis by examining the association between parental support and psychological complaints. Models 2–3 intend to answer the second hypothesis by adding potential confounders at the individual level (Model 2) and at the school level (Model 3). Finally, Model 4 examines the mediating role of school climate, while also adjusting for all potential confounders, in order to answer the last and third hypothesis. In order to assess the model improvement between the models, the Likelihood-ratio test was performed, where each model is compared to the previous one.

The mediation analysis is based on Baron and Kenny’s four-step mediation model [43]. First, step 1 examines whether the independent variable is significantly associated with the dependent variable, followed by step 2, which ensures that the independent variable is significantly related to the assumed mediator. The third step examines whether the mediating variable is significantly related to the dependent variable. Finally, the fourth step is about confirming that the previously significant association between the independent variable and the dependent variable decreases (or turns out to be non-significant) when the expected mediator is added to the model. In other words, if the association between the independent variable and the dependent variable decreases when the mediator is included in the model, it indicates a mediating effect [43]. Additionally, Sobel’s test was used to test the significance of a mediation effect. The first three steps in the mediation analysis are performed through bivariate associations, while the fourth and last step is examined in Model 4 of the regression analysis.

## 3. Results

Table 2 presents descriptive statistics for all variables used in the analyses. The mean value for the dependent variable psychological complaints is 13.9, in the range 6–30. The independent variable, parental support, is in the range 5–20 and the mean value is 16.7. The mean grade for the three subjects Swedish, English, and mathematics is 8.9, and there are approximately the same number of girls as boys in the study sample. About two-thirds of the students report that they live with two parents and about 58 percent state that at least one of their parents has a post-secondary education. The vast majority, almost 93 percent, have lived in Sweden for ten years or longer. At the school level, 87 percent of the students attend a public school. The mean value of the proportion of full-time teachers per student is 14.2, while the mean value of the proportion of full-time teachers with a pedagogical degree is about 85 percent. About one quarter of the students in the study sample have a foreign background, i.e., they were born abroad and/or both parents were born abroad. Finally, the potential mediating variable, the school climate index, is approximately normally distributed, in the range 18–72, with a mean value of 49.0.

The first column on the right side of Table 2 shows the bivariate associations between the dependent variable and all other variables used. It can be noted that the association between parental support and psychological complaints is negative and statistically significant (b = −0.31 *p* < 0.001), thus confirming the first step in Baron and Kenny’s [43] model for testing mediation. It can also be noted that girls report significantly higher levels of psychological complaints than boys do (b = 0.37 *p* < 0.001), and that living with two parents is significantly related to lower levels of psychological complaints compared to living with one parent (b = −0.10 *p* < 0.001). The second column on the right side of Table 2 reports the bivariate associations between school climate and all variables used. Of most interest to the model for testing mediation, school climate is positive and statistically significantly associated with parental support (b = 0.33 *p* < 0.001), which confirms step two in the mediation analysis. Furthermore, school climate is negative and statistically significantly associated with psychological complaints (b = −0.36 *p* < 0.001), thus confirming step three in the model for testing mediation.

It can also be noted that school climate is positively associated with grades and that boys rate the school climate somewhat higher than girls do. Taken together, from the bivariate analysis, it can be concluded that the first three steps in the model for testing mediation have been fulfilled, namely that the independent variable (parental support) is significantly related to the dependent variable (psychological complaints) and the potential mediator (school climate), just as the potential mediator is significantly related to the dependent variable.

Next, the linear regression analyses were performed in several steps, and presented in a series of four models, as shown in Table 3. The first model shows the association between parental support and students’ psychological complaints (−0.52, *p* < 0.001), indicating that students’ average ratings on psychological complaints lie 0.52 units lower for every step in the parental support index. The second model adds the potential confounders at the individual level, and it can be seen that the association between parental support and psychological complaints is not affected to any noteworthy degree. It can also be noted that girls report significantly higher levels of psychological complaints than boys do, and also that living with two parents is related to lower levels of psychological complaints than living with only one parent. In Model 3, the potential confounders at the school level are taken into account, but the association between parental support and psychological complaints is not affected here either, but rather remains robust and statistically significant at the same level.

Finally, in Model 4, the potential mediating variable of school climate is added, and it can be seen that school climate has an impact on students’ psychological complaints (–0.16, *p* < 0.001). At the same time, the estimate of parental support increases to –0.40, indicating a mediating effect of school climate. Furthermore, the indirect effect and the results of Sobel’s test were significant (z = −17.19, *p* < 0.001). Thus, it can be concluded that school climate has a partly mediating role in the association between parental support and students’ psychological complaints. This is further supported by the Likelihood-ratio test showing a significant model improvement when school climate is added to the analysis.

## 4. Discussion

Since as many as up to one-fifth of all children and adolescents, around the world, experience mental health problems [3], it is a very important task to investigate possible protective factors and environments. It has already been well-established in previous research that parental support is an important factor [9,26,27,28,29,30,31,32], not only for younger children but also for adolescents [33,34]. It has also been well-established in previous research that the school climate is of great importance for the well-being of children and adolescents and for a number of other important aspects [38,39,40,41]. On the other hand, there have been few studies that have examined possible mediating effects that the school climate may have [16].

The aim of this study was to examine the association between parental support and psychological complaints, as rated by students, with a particular interest in the possible mediating role that a good school climate may have.

The first hypothesis of the study concerned whether students’ ratings of parental support were associated with psychological complaints. The results reveal a clear negative significant association between parental support and psychological complaints, where students who rate the support from their parents as higher also express lower levels of psychological complaints, and vice versa. This result confirms the study’s first hypothesis and is in line with an overall picture of international research that has previously emphasized the importance of parental support for the mental health and well-being of children and adolescents [9,26,27,28,29,30,31,32]. More specifically, previous studies [30,31,32] have shown that perceived low parental support is negatively associated with psychological complaints, just as shown in this study.

The second hypothesis of the study concerned whether this association remained when adjusting for a number of variables at the individual and the school levels. The results show that the control variables included had no noticeable effect on the relationship between parental support and psychological complaints, which confirms the study’s second hypothesis. It can also be noted that girls in this study sample reported higher levels of psychological complaints than boys did, also in line with previous international research [1,2,4]. Furthermore, it could also be concluded that adolescents living with two parents in the same household reported lower levels of psychological complaints than adolescents living with only one parent. It is reasonable to assume that living with two parents means increased opportunities to experience support from parents, compared to living with just one parent.

The third and the study’s main hypothesis was about whether a good school climate serves as a mediator in the association between parental support and psychological complaints. As the importance of parental support decreased when school climate was introduced into the analysis, it can be concluded that the association between parental support and psychological complaints is partly mediated by a good school climate, thus indicating that the third hypothesis can be partly accepted. This result is further supported by Sobel’s test, which revealed a significant mediation effect of school climate. This means the study contributes new knowledge as there has previously been a lack of studies that have examined the school climate’s potential mediating effects [16]. One way to understand this partly mediating effect may be that students who experience low parental support seek other contexts, such as school, to receive this support. In other words, the school climate may to some extent compensate for a low parental support. It can also be understood as the support offered at the school to some extent replaces the support from parents, although previous research has indicated that support from parents and the school are to some extent independent sources of support [13,14]. Results from this study, indicating that the school climate is of importance for young people’s well-being and health, are very much in line with previous international research, showing for example that a good school climate is associated with a positive psychological development [38], and improved mental health outcomes [15,41].

As it can be concluded that the school climate has an impact on students’ well-being and psychological complaints, educational policies and guidelines should to a greater extent include efforts to strengthen the school climate in order to help counteract mental health problems and increase the well-being of young people. Strengthening the school climate can be done, for example, through initiatives that clarify the school’s regulations and rules of procedure, to strengthen the school’s working methods for praise and support from teachers to students, as well as increase students’ involvement in decision-making, and to develop the working environment and safety in school.

### Strengths and Limitations

One strength of this study is the relative high response rate (82.7%) among students. However, it is reasonable to assume that students that did not attend school while the survey was conducted would have reported higher levels of psychological complaints, lower levels of parental support, and the school climate as worse, but there is no reason to believe that such a potential non-response bias would have affected the relationships investigated. Another strength is the possibility to adjust for a number of potential confounders at the individual level, as well as the opportunity to adjust for potential confounders at the school level through the access to official records from the SNAE. The study also has some limitations to take into account. First, the study is built on data from senior-level school students in Stockholm municipality, which means that generalizations to other geographical frameworks and age groups must be made with caution. Secondly, since the data used for the study are of a cross-sectional nature, it is not possible to draw any conclusions about causality. Longitudinal data is desirable for future studies. Finally, since students who did not answer all items related to this study were excluded, there is a possibility that there is some selection bias of students.

## 5. Conclusions

It can be concluded that there is a negative significant association between parental support and students’ psychological complaints, but that attending a school with a good school climate may reduce the effects of poor parental support. Therefore, it seems important not only to target efforts to increase parental support, but also to invest in developing a good school climate to reduce students’ experiences of psychological complaints. This could be especially important in school areas where students come from homes with generally poor parental support.

## Figures and Tables

**Table 1:** Items included in the dependent, independent, and mediating variables.

**Psychological Complaints**	**Cronbach’s Alpha 0.8008. Range 6–30.** **6 = Less Psychological Complaints,** **30 = More Psychological Complaints.**
Original questions:	Response options:
Do you feel sad and depressed without knowing why?	1 = seldom 2 = occasionally 3 = sometimes 4 = pretty often 5 = very often
Do you ever feel frightened without knowing why?	1 = seldom 2 = occasionally 3 = sometimes 4 = pretty often 5 = very often
Do you feel sluggish and uneasy?	1 = seldom 2 = occasionally 3 = sometimes 4 = pretty often 5 = very often
How often do you feel it’s really good to be alive? (reversely coded)	1 = seldom 2 = occasionally 3 = sometimes 4 = pretty often 5 = very often
How much would you like to change yourself?	1 = not at all 2 = not much 3 = some 4 = pretty much 5 = very much
How often do you feel you’re not good enough?	1 = almost never 2 = occasionally 3 = sometimes 4 = pretty often 5 = very often
**Parental support**	**Cronbach’s alpha 0.8176. Range 5–20.** **5 = less parental support,** **20 = more parental support.**
Original question: How well do the following statements describe your parents’/guardians’ relationship with you?	Response option: 1 = describes very poorly 2 = describes rather poorly 3 = describes rather well 4 = describes very well
They praise me when I do something good	
They usually encourage and support me	
They notice when I do something good	
I care about what my parents/guardians say	
My parents/guardians are an example to me	
**School climate**	**Cronbach’s alpha 0.8356. Range 18–72.** **18 = poor climate, 72 = good climate.**
Original question: How well do the following statements describe your situation at school?	Response option: 1 = describes very poorly 2 = describes rather poorly 3 = describes rather well 4 = describes very well
I am aware of the regulations that apply at this school	
I enjoy going to school	
Students take part in the planning of what we will do in class	
Teachers praise students who do something good at school	
There’s a lot of noise and rowdiness in class (reversely coded)	
Schoolwork feels pointless (reversely coded)	
Students take part in making decisions on things that are important to us	
The teachers let us know what we can and can’t do	
When a class starts it takes at least five minutes before we can get started	
The school lets my parents know if I’ve done something good	
Students’ views are not taken seriously at this school	
Adults step in if anyone is harassed or bullied	
My teacher doesn’t give me any praise when I work hard (reversely coded)	
I look forward to going to my classes	
I’m worried about being subjected to crime at school	
Most of my teachers make learning interesting	
Schoolwork makes me confused (reversely coded)	
If you don’t understand something, you get help from the teacher straight away	

**Table 2:** Descriptive statistics and bivariate associations of all variables included in the analyses. *n* = 5783 students across 152 school units.

				Bivariate Associations with Psychological Complaints	Bivariate Associations with School Climate
*Dependent variable*	Mean	SD	Range		
Psychological complaints	13.9	5.1	6–30		−0.36 ***
*Independent variable*					
Parental support	16.7	3.0	5–20	−0.31 ***	0.33 ***
*Potential confounders, student-level*					
Grades	8.9	3.6	0–15	−0.02	0.15 ***
Gender	*n*	%			
-Boys (ref.)	2834	49.0		0.00	0.00
-Girls	2949	51.0		0.37 ***	−0.11 ***
Family structure					
-Living with one parent (ref.)	1883	32.6		0.00	0.00
-Living with two parents	3900	67.4		−0.10 ***	0.04 **
Parental education					
-No parent with post-secondary education (ref.)	2443	42.2		0.00	0.00
-At least one parent with post-secondary education	3340	57.8		0.01	0.04 *
Time in Sweden					
-Less than ten years (ref.)	425	7.4		0.00	0.00
-Ten years or more	5358	92.6		−0.01	−0.04 **
Year					
-2014	2771	47.9		0.00	0.00
-2016	3012	52.1		−0.01	0.01
*Potential confounders, school-level*					
School type	*n*	%			
-Public (ref.)	5032	87.0		0.00	0.00
-Independent	751	13.0		0.01	0.07 ***
	Mean	SD	Range		
Proportion of full-time teachers per student	14.2	2.6	7.6–23.7	−0.02	−0.03 *
Proportion of full-time teachers with pedagogical education	84.7	8.3	33–100	0.00	−0.02
Proportion of students with foreign background	27.7	23.5	5–98	−0.00	−0.04 **
*Potential mediator, individual-level*					
School climate	49.0	7.9	18–72	−0.36 ***	

*** *p* < 0.001; ** *p* < 0.01; * *p* < 0.05.

**Table 3:** Linear regression analyses of psychological complaints (*n* = 5783 students distributed over 152 schools).

	Model 1		Model 2		Model 3		Model 4	
	Coef.	95% CI	Coef.	95% CI	Coef.	95% CI	Coef.	95% CI
Parental support	−0.52 ***	−0.56–−0.48	−0.54 ***	−0.57–−0.50	−0.54 ***	−0.57–−0.50	−0.40 ***	−0.44–−0.36
Grades			−0.03		−0.04 *		0.01	
Gender			3.91 ***		3.93 ***		3.55 ***	
Family structure			−0.58 ***		−0.58 ***		−0.60 ***	
Parental education			0.09		0.04		0.07	
Time in Sweden			0.25		0.17		−0.27	
Year			0.00		0.00		−0.02	
School type					−0.10		0.19	
Proportion of full-time teachers per student					−0.07 *		−0.11 **	
Proportion of full-time teachers with pedagogical education					0.00		0.00	
Proportion of students with foreign background					−0.01 **		−0.01 ***	
School climate							−0.16 ***	−0.15–−0.15
Model improvement			*p* < 0.001		*p* = 0.043		*p* < 0.001	

*** *p* < 0.001; ** *p* < 0.01; * *p* < 0.05.
